# Effects of Low-Dose Carbon Monoxide on Antibiotic Efficacy

**DOI:** 10.17912/micropub.biology.001800

**Published:** 2025-12-18

**Authors:** Alana White, Yasmeen Rasasi, Stuart Gordon, Ladie Kimberly De La Cruz

**Affiliations:** 1 Department of Chemistry and Biochemistry, Presbyterian College; 2 Department of Biology, Presbyterian College

## Abstract

The impact of intestinal gases, including gasotransmitters, on antibiotic efficacy is severely understudied. This study assessed the effects of low-dose CO (85 µg/g) on the efficacy of various antibiotics in low oxygen conditions using the Kirby-Bauer method against *E. coli* BW20767/pRL27. Preliminary results showed that exposure to CO exerts variable effects on antibiotic efficacy. This indicates that CO exerts its effects not only through modulation of *E. coli* ’s respiratory chain, but may also involve additional, as-yet unidentified targets independent of terminal oxidase binding. We also observed that *E. coli* endogenously produces CO as it switches to anaerobic metabolism.

**Figure 1. Exposure to low-level CO exerts variable effects on antibiotic efficacy against E. coli grown under hypoxic conditions:**
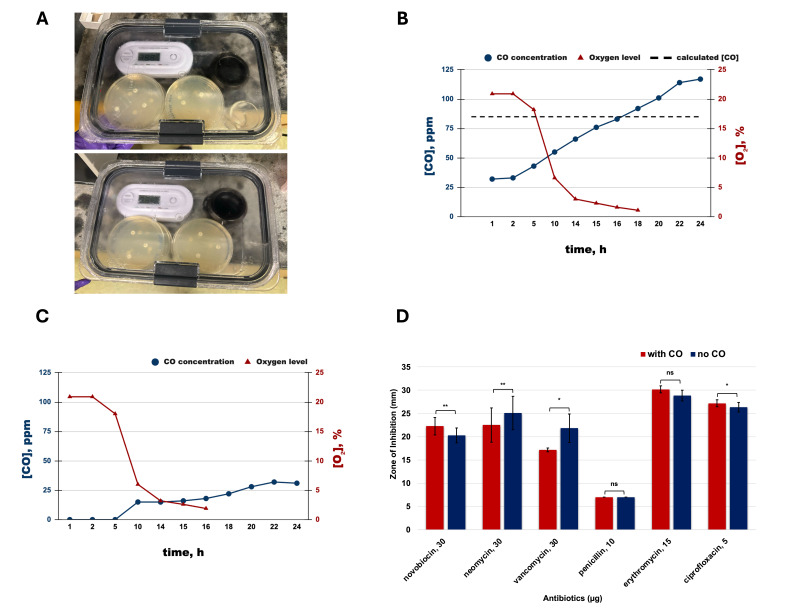
(A) Experimental set-up of closed, hypoxic chambers equipped with oxalyl chloride as the external CO source, CO detector, and O _2_ detector. O _2_ and CO levels in the sealed chambers containing: (B) externally generated CO up to 85 ppm and (C) ambient air. (C) Average zones of inhibition (mm) via Kirby Bauer method for various antibiotics with and without CO exposure (technical replicates = 6, independent replicates: novobiocin = 3, neomycin = 2, vancomycin = 1, penicillin = 2, erythromycin = 1, ciprofloxacin = 1). Error bars indicate ± standard deviation. * p < 0.05, ** p < 0.01, ns - not significant. &nbsp;

## Description

Intestinal gas in humans is primarily composed of nitrogen (65%), methane (14.4%), carbon dioxide (9.9%), hydrogen (3%), and oxygen (2.3%) (Tozzi and Minella, 2024). While these major gases contribute to the overall volume and physical properties of intestinal gas, a minor but biologically significant fraction consists of gasotransmitters such as hydrogen sulfide (H _2_ S), nitric oxide (NO), and carbon monoxide (CO). The production of these gasotransmitters is tightly regulated by both host cells and the gut microbiota, highlighting their dual origin and essential signaling functions within the gut (Hopper, et. al., 2020). Intestinal gases produced by both the host and the microbiome are not merely waste products but play essential roles as biological mediators in energy metabolism, bacterial proliferation, and other key physiological processes (Tozzi and Minella, 2024). While diet is a major factor influencing the intestinal gas profile by modulation of the gut microbiome (Modesto et. al, 2021), antibiotics can also profoundly alter the gut microbiota by reducing microbial diversity and selectively eliminating certain bacterial populations (Dethlefsen and Relman, 2011). These shifts in microbial composition can disrupt normal fermentation patterns and metabolic activity, leading to changes in types and volumes of gases produced within the intestines (Stefano, et. al., 2021; Sobko, et. al., 2007). Given that modulation of the gut microbiome can significantly influence intestinal gas volume and composition, there is growing interest in exploring the potential bidirectional nature of this relationship, particularly in understanding how exposure to varying levels of gasotransmitters may affect microbial physiology and in turn, potentially alter antibiotic efficacy.

*E. coli* exposed to 25% CO gas altered the activity of key transcription factors that regulate metabolism and stress responses (Wareham et. al, 2016). Furthermore, CO was found to inhibit *bd* -type oxidases in the electron transport chain that disrupted respiration, and triggered the upregulation of glycolysis and NADH dehydrogenases to maintain ATP production. The study also explored the effects of 25% CO on the efficacy of three antibiotics with different mechanisms of action: doxycycline, trimethoprim, and cefotaxime. The findings revealed that CO exposure enhanced the efficacy of the antibiotics in aerobic and anaerobic conditions. Building on these CO gas-based studies, we envisioned to study the effects of CO at a concentration that is representative of the gaseous environment of the human gut of ≤1% CO under low-oxygen conditions. This study investigated the susceptibility of *E. coli* to a range of antibiotics and evaluated the potential of low-dose CO as an antibacterial adjuvant. Six antibiotics were tested using the Kirby-Bauer assay, including novobiocin, neomycin, erythromycin, penicillin, vancomycin, and ciprofloxacin. The selection of these antibiotics was based on their distinct modes of antibacterial action, specifically targeting protein synthesis, nucleic acid synthesis, and cell wall synthesis. To ensure controlled CO exposure, two airtight chambers were developed: one for CO treatment and another as a no-CO control (Figure A). Preliminary experiments explored and optimized oxalyl chloride, (COCl) _2_ , as the most economical and suitable method to deliver the gas into the chamber. CO generation was achieved via the chemical reaction between oxalyl chloride and sodium hydroxide (Hansen et. al., 2015). The reaction was conducted in the presence of a base to trap and convert carbon dioxide and neutralize acids to sodium salts, producing CO as the main gaseous product. The reaction proceeds as follows: (COCl) _2_ + 4NaOH → CO + Na _2_ CO _3_ + 2NaCl + 2H _2_ O. To ensure controlled release of CO, we carefully placed a 2-mL vial containing 2 μL oxalyl chloride inside a 50-mL beaker with 10 mL 2M NaOH. To initiate CO release, gentle agitation of the sealed chamber would jumpstart the reaction and generate approximately 85 µg/g or ppm of CO. Before initiating the reaction, six agar plates inoculated with *E. coli* and charged with antibiotic disks together with CO and oxygen detectors were placed into the chamber. The chamber was sealed with vacuum grease to ensure gas containment, and gentle agitation was applied to initiate CO release. Gas levels were then monitored periodically over the 24-hour incubation period. Another chamber was prepared with an identical setup, excluding the internal vial of oxalyl chloride and the beaker of sodium hydroxide to serve as the control. Zones of inhibition were then measured to assess antibiotic efficacy under both conditions.

In the chamber containing the exogenous CO source, CO levels rose steadily from 31 ppm to 117 ppm over 24 hours, while O _2_ dropped rapidly from 20.9% to 1.1% by 18 hours. As oxygen was consumed by *E. coli* to support metabolic activity, CO levels continued to rise due to ongoing chemical generation and no active removal of CO. However, CO levels in this chamber exceeded the calculated threshold of 85 ppm by 32 ppm at the 24-h timepoint (Figure B). Notably, the control (no-CO) chamber unexpectedly exhibited CO production after 10 hours, reaching 31 ppm by 24 hours (Figure C). The observed CO production also coincided with oxygen levels of around 5% indicating a link between endogenous CO generation and metabolic shift to fermentation/anaerobic metabolism. While it is established that host cells via heme oxygenase (HO) generate CO as a cytoprotective and stress response to invading *E. coli* infection (Chen, et. al, 2006; Weigel, et. al, 2014), there are few reports regarding endogenous production of CO by *E. coli* itself. ChuS, a non homologous HO-like enzyme was reported to degrade heme and release CO with H _2_ O _2_ as the electron source instead of molecular O _2_ (Ouellet, et. al., 2016). Another possible endogenous source of CO production in *E. coli* is through non-enzymatic auto-oxidation of the porphyrin ring in heme.

Among the antibiotics evaluated, novobiocin (30 μg) and ciprofloxacin (5 μg) which are both DNA gyrase inhibitors, albeit via distinct mechanisms, demonstrated a statistically significant enhancement in efficacy when combined with CO, whereas neomycin (30 μg), and vancomycin (30 μg) showed a statistically significant reduction in effectiveness in the presence of CO. On the other hand, antibiotics such as penicillin (10 μg) and erythromycin (15 μg) did not exhibit any significant changes in efficacy upon exposure to low-dose CO. In *E. coli* , heme proteins such as the terminal oxidases of respiratory chains are the most likely CO targets (Nastasi et. al., 2023; Borisov and Forte, 2025). However, the results indicate that CO’s effect on antibiotic efficacy is dependent on the specific mechanism of action of the antibiotic, rather than exhibiting uniform effectiveness across diverse antibiotic classes. These preliminary findings warrant further investigation on the link between CO and DNA gyrase inhibitor-based antibiotics for potential adjuvant antibiotic therapy. Furthermore, the results highlight the role of baseline intestinal gas profiles, including levels of gasotransmitters such as CO, in influencing antibiotic treatment outcomes.

## Methods

Bacterial cell lines and culture conditions

*Escherichia coli* BW20767/pRL27 (Larsen, et. al., 2002) was cultured on Luria broth (LB) agar plates prepared following standard protocols and stored at 4°C. For experiments, approximately four isolated colonies were inoculated separately into 1 mL LB broth in two microcentrifuge tubes and incubated overnight at 37°C with agitation (190 rpm). Cultures were centrifuged at 6,000 rpm for 1 minute, supernatants discarded, and bacterial pellets resuspended in sterile saline. The suspensions were combined and adjusted to a turbidity equivalent to the 0.5 McFarland standard (~1.5 x 10 ^8^ CFU/mL) by visual comparison against a reference standard.

Chamber Preparation

Airtight 2.3 L plastic chambers were utilized to contain the agar plates along with CO and oxygen sensors, which were routinely calibrated and verified prior to experimentation. To generate approximately 85 ppm CO, 2 μL of oxalyl chloride suspended in 10 mL of 2 M sodium hydroxide was placed inside the chamber. The container was sealed using vacuum grease and gently agitated to initiate CO production. Control chambers were assembled using the same procedure, but without the addition of oxalyl chloride and sodium hydroxide.

Kirby-Bauer Disk Diffusion Assay

A 100 μL aliquot of the standardized cell suspension was plated onto LB agar and evenly distributed using a metal spreader sterilized between plates by immersion in ethanol followed by flaming, and allowed to cool briefly prior to use. The remaining suspension was resuspended between aliquots to ensure consistent density across all plates.

Antibiotic susceptibility was evaluated using the Kirby-Bauer disk diffusion method with six antibiotics: novobiocin (30 μg), neomycin (30 μg), penicillin (10 μg), vancomycin (30 μg), erythromycin (15 μg), and ciprofloxacin (5 μg). Antibiotic disks were applied using a disk dispenser to ensure uniform placement and consistent contact with the agar surface.

Three antibiotics were tested in each experimental set under two atmospheric conditions: CO-exposed and ambient air. For each gas exposure-antibiotic combination, six replicates were performed, totaling 12 plates per experiment. Each plate was labeled with the date, antibiotic combination, experimental condition, and research initials. Antibiotic disks were stored at 4°C prior to use to maintain potency.
